# Supplementary material for: Dietary Dihydroartemisinin Supplementation Attenuates Hepatic Oxidative Damage of Weaned Piglets with Intrauterine Growth Retardation through the Nrf2/ARE Signaling Pathway
Source: Animals (Basel). 2019 Dec 13;9(12):1144. doi: 10.3390/ani9121144 (PMC6941019; doi:10.3390/ani9121144)
Supplement: Supplementary file 1 [file animals-09-01144-s001.pdf]

animals

Supplementary Materials: Dietary Dihydroartemisinin Supplementation Attenuates Hepatic Oxidative Damage of Weaned Piglets with Intrauterine Growth Retardation through the Nrf2/ARE Signaling Pathway

Yongwei Zhao, Yu Niu, Jintian He, Lili Zhang, Chao Wang and Tian Wang *

College of Animal Science and Technology, Nanjing Agricultural University, No. 6, Tongwei Road, Xuanwu District, Nanjing 210095, China; zhaoyongweinjau@163.com (Y.Z.); niuyu0227@126.com (Y.N.); 15150537273@163.com (J.H.); zhanglili@njau.edu.cn (L.Z.); wangchao121@njau.edu.cn (C.W.)

* Correspondence: tianwangnjau@163.com; Tel./Fax: +86-025-84395156

Table S1. Effects of dietary dihydroartemisinin supplementation on growth performance of IUGR weaned piglets.

**Item** †	**Experiment Groups**		
	**NBW**	**IUGR**	**IUGR-DHA**
BW (kg): 21 days	6.96 ± 0.05 [a]	6.08 ± 0.09 [b]	6.08 ± 0.06 [b]
BW (kg): 49 days	12.80 ± 0.60 [a]	10.24 ± 0.38 [b]	12.11 ± 0.46 [a]
BWG (kg)	5.84 ± 0.62 [a]	4.17 ± 0.35 [b]	6.03 ± 0.49 [a]
FI (kg)	8.00 ± 0.13 [b]	6.55 ± 0.15 [c]	9.13 ± 0.13 [a]
G:F (kg/kg)	0.73 ± 0.08	0.64 ± 0.06	0.66 ± 0.06

[a,b,c] Mean values within a row with unlike superscript letters were significantly different ($p < 0.05$). Values were means and standard errors ($n = 8$). † NBW, normal birth weight group given a basal diet; IUGR, Intrauterine growth retardation group given a basal diet; IUGR-DHA, IUGR group given diets supplemented with 80 mg/kg dihydroartemisinin; BW, body weight; BWG, body weight gain; FI, feed intake; G:F, body weight gain: feed intake.
